# Challenges of Assessing Maltreated Children Coming into Foster Care

**DOI:** 10.1155/2016/5986835

**Published:** 2016-01-06

**Authors:** Rachel Pritchett, Harriet Hockaday, Beatrice Anderson, Claire Davidson, Christopher Gillberg, Helen Minnis

**Affiliations:** ^1^Institute of Health and Wellbeing College of Medical, Veterinary and Life Sciences Academic Unit of Mental Health & Wellbeing, University of Glasgow Caledonia House Royal Hospital for Sick Children Yorkhill, Glasgow G3 8SJ, UK; ^2^NSPCC Scotland, Glasgow Service Centre, Pavillion 2, Rowan Business Park, 5 Ardlaw Street, Glasgow G51 3RR, UK; ^3^Gillberg Neuropsychiatry Centre, University of Gothenburg, Kungsgatan 12, 411 19 Gothenburg, Sweden

## Abstract

Children who have experienced early adversity have been known to be at risk of developing cognitive, attachment, and mental health problems; therefore, it is crucial that children entering foster care can be properly assessed as early as possible. There are known difficulties in assessing children in foster care, for example, in finding a reliable informant. An ongoing randomised controlled trial in Glasgow, Scotland, recruiting infants entering foster care, provides a unique opportunity to explore some of the issues which need to be considered when assessing these children. The assessment data of 70 infants entering care is described while exploring the reliability of foster carers as informants and the importance of infant engagement with tasks. This group of infants was shown to be having more problems than children from the general population. While correlations were found between a carer's level of concern about a child and the severity of a child's problem, there were still a number of children displaying worrying problem scores whom foster carers did not report concern. The child's engagement in the cognitive task showed associations with the child's attainment on the task. Findings emphasise the importance of a holistic assessment for these children and all should be considered as potential cases with Maltreatment-Associated Psychiatric Problems (MAPP).

## 1. Introduction

It is now well established that early childhood adverse experiences are strongly associated with risk of developing problems in later life [1]: in terms of later health [2], social development [3], mental health and wellbeing [4], and educational attainment [5].

Children entering foster care are at high risk of having experienced considerable early adversity and demonstrate high levels of psychopathology, educational difficulties, and neurodevelopmental disorders compared to peers reared at home [5–7]. Children in foster care may also have a higher prevalence of attachment disturbances; Smyke et al. [8] found higher rates of Reactive Attachment Disorder (RAD) in institutionalised children compared with those who had never been institutionalised. RAD is a severe disorder of social functioning thought to be caused by early maltreatment. It is persistent [9] and is associated with significant psychiatric morbidity [10, 11], suggesting increased vulnerability of these children, in both early childhood and the future.

Because of the clearly increased risk of children in foster care developing later problems, it is crucial that these children's needs are assessed accurately and as early as possible, for example, to ensure that interventions can be implemented quickly. Clinicians are often required to make assessments of children when they first enter care, and the child's apparent profile of needs may have an effect on where they are placed and the support they receive. Several studies have assessed children when they first come into foster care [6, 12]; however, the authors of these studies also acknowledge the difficulties in assessing this young and vulnerable group at such a turbulent time.

Historically, foster parents' perceptions of difficulties were seen as a cornerstone of assessing the child's needs: “it is the foster parents' perceptions of the seriousness of the problem that are all important” [13]. More recent experts in the field have taken a different stance, suggesting that the foster carer report alone is not sufficient. Carter et al. [14] identified a number of challenges related to assessing infants in care. These include contextual influences, child behaviour, overlap between problems, problems finding reliable informants, and the difficulty of symptoms being indicative of more than one domain; for example, a child may score lacking self-control, but this could in fact be a reflection of global developmental delay. As there is still a reluctance to identify mental health problems in very young children, caregivers are sometimes unable to distinguish between normative behaviours and clinically concerning behaviours. This makes it difficult when trying to detect problems early. A child's birth parent may be able to provide information about the child's former or usual presentation, but in the legally and emotionally fraught period following the child's accommodation, they may not be reliable informants. Also, there are often multiple challenges in their own lives that may impact the ability to provide an objective, valid assessment. It is crucial, therefore, to use multiple approaches and informants [14].

Minnis [15] recently described a new concept: Maltreatment-Associated Psychiatric Problems (MAPP), a syndrome of overlapping complex neurodevelopmental problems in children who have experienced abuse or neglect in early life. Minnis argues that the early life events these children face place them at an increased risk of developing problems and that when problems do arise in the context of maltreatment, they are likely to be complex and overlapping.

While it seems important to interpret social and emotional problems in line with what is known about the child's cognitive development, it should be noted that cognitive ability may not be a stable measure in these children. O'Connor et al. [16, 17] demonstrated “developmental catch-up” following adoption of Romanian orphans placed into UK homes, in which young children placed in the UK significantly increased their cognitive scores when they were followed up at the age of 6 years.

When assessing the mental health of a young child who has been accommodated recently, assessments may reflect an especially transitory picture, due to active processes of change and the recent trauma that the child may have experienced in the move from their birth parents. Furthermore, when relying on caregivers who have not known the child long to provide information, it may not be possible to gain a full perspective on the child's state over the period specified by assessment measures, or of how current behaviour compares with his/her usual functioning.

A recently accommodated child has just been through a major life event (loss of primary caregivers) and is subject to processes of adjustment, with associated emotional and behavioural sequelae, such as despair, crying, and aggression [18]. Best practice guidelines for Posttraumatic Stress Disorder [19] note that particularly traumatic events are those likely to cause “pervasive distress in almost anyone” and recommend* watchful waiting* in situations where symptoms are mild and have been present for less than four weeks following a traumatic event. However, a good understanding of the child's current difficulties may allow appropriate supports to be put in place, for example, to prevent foster placement disruption.

Accommodated children also endure attachment disruption [20]; Bowlby described children move from protesting the separation from primary caregivers to despairing and losing hope of reunion, and finally reattaching to an available alternative caregiver. A variety of factors can impact the speed and quality of this process, such as the child's age, previous experiences, and resiliencies, as well as the quality of alternative caregiving and any ongoing contact with original caregivers [21].

We were interested in describing the results of our assessments conducted with children entering foster care as well as some data which aims to disentangle some of the issues which need to be considered when assessing children when they enter foster care.

## 2. Method

### 2.1. Participants

Participants were 70 children aged 6–60 months (mean age of 34 months) who entered a period of foster care due to child protection concerns.

### 2.2. Measures

#### 2.2.1. Bayley Scales of Infant and Toddler Development (BSID-III)

The Bayley scales are used to measure different aspects of child's development [22, 23], by engaging them in developmental play tasks. In this study, the Bayley scales were used with children aged 12 to 29 months and the children were assessed on the cognitive and language components of the measures. The Bayley scales ask the administrator to rate how easy it was to engage the child in the tasks, reporting no difficulty in engagement, some difficulty, or a lot of difficulties. Following initial recruitment, the benefits of this observation were noticed and so it was decided to also complete these ratings with children engaging in the WPPSI. The decision to add this measure came after the start of these assessments, however, so this data is only available for 56 of our children.

#### 2.2.2. Wechsler Preschool and Primary Scale of Intelligence (WPPSI 3rd Edition)

The WPPSI is a scale of intelligence producing both an “intelligence quotient” (IQ) and scaled scores by age [24, 25]. It contains 10 subtests, five of which measure performance IQ and 5 measure verbal IQ, with a full scale IQ produced when these are combined. The WPPSI has been shown to be a good measure of general intelligence producing reliable and stable IQs and was used with children aged 30–60 months in this study.

#### 2.2.3. Infant-Toddler Social Emotional Assessment (ITSEA)

The ITSEA is a 166-item questionnaire which is completed by the primary caregiver [26]. It provides an assessment of the child's social and emotional development and any behavioural delays over 4 domains: externalising, internalising, dysregulation, and competence. It also includes questions regarding the degree of worry which the carer has about the child, in terms of both their development and their mental health. In this study, it is used to describe the children aged 12–48 months, covering the age range for which there is normative data available [27].

#### 2.2.4. Strengths and Difficulties Questionnaire (SDQ)

The Strengths and Difficulties Questionnaire (SDQ) is a 25-item screening questionnaire which investigates common mental health problems in children and has been well validated against other screening instruments [28] and against psychiatric diagnosis [29]. It covers 5 domains, with 5 questions on each of the following areas: emotional problems, conduct problems, hyperactivity, problems with peer relationships, and prosocial behaviour (caring, helpful behaviour). In this study, it was used to describe the children aged over 24 months.

#### 2.2.5. Development and Wellbeing Assessment (DAWBA)

The DAWBA [30] is a screening questionnaire for a number of psychiatric diagnoses. It covers a wide range of disorders including emotional, behaviour, and hyperactivity disorders. It can be used with caregivers of children between the ages of 2 and 17 and was used with every child over the age of 2 in this study. The DAWBA can be completed either using a paper format or, as in this study, using a computerised format. The DAWBA has been shown to be a valid measure of child psychopathology [30] and has been used in nationwide surveys of child and adolescent mental health [31].

#### 2.2.6. Disturbances of Attachment Interview (DAI)

The DAI is a 12-item semistructured interview which is administered by clinicians to a child's caregiver. The DAI is made up of 3 sections which cover disinhibited behaviours, inhibited behaviours, and secure base distortions. Responses to each of the 12 items are coded on a three-point scale: clearly demonstrating a behaviour, sometimes or somewhat demonstrating a behaviour, and rarely or never demonstrating a behaviour. The DAI scales have demonstrated strong internal validity (Cronbach *∝* 0.83 and 0.80, resp.) and excellent interrater reliability (*κ* = 0.88) [32].

#### 2.2.7. Parents' Evaluation of Developmental Status (PEDS)

The PEDS is a developmental screening test which can be used with caregivers of children aged 0–8 years [33]. Caregivers are asked to report on their level of concern across different areas of the child's development. The PEDS results in the children being classified as at high, moderate, or low risk of developing problems [34]. The PEDS has been shown to have moderate sensitivity (0.79) and specificity (0.80) [35]. The PEDS was used with every child in the study.

### 2.3. Procedure

Recruitment was carried out through a larger randomised controlled trial (RCT) testing the effects on child mental health of a mental health intervention for families with a young child coming into foster care [36]. Following the child coming into care, a period of at least 4 weeks was allowed to elapse before baseline assessment for the carer to get to know the child and for the child to “settle into” their new home. For the purposes of the trial, follow-up assessments were also carried out, but these are not described here.

The child and foster carer were invited to attend the clinic where the child was assessed using the Bayley scales if they were under 30 months and the WPPSI if they were over 30 months. A researcher then completed the ITSEA and the DAI with the foster carers if the child was over 12 months and also the SDQ and DAWBA if the child was aged over 24 months.

The results describe the outcomes of the assessments with the 70 children, comparing them with data from normative samples collected in other studies. In addition, we investigated the reliability of foster carers as informants by examining the relationship between the carers level of worry about a child (as measured on the ITSEA and PEDS) and the child's ability in that area. Finally, we investigated the extent to which the child's level of engagement, as measured by an observational checklist, affected the child's performance on a task.

## 3. Results

### 3.1. Sample Demographics

The sample mean age was 34 months (range of 8 to 62 months) with 41 males (59%). Sixty-two (89%) of the children in the sample were white Scottish, 2 (3%) were other British, 3 (4%) were Pakistani, and 3 (4%) were African (2 Black African, 1 other African).

### 3.2. Development

On the Parent Evaluation of Developmental Status (PEDS), the majority of children were described as having “no” or “a little” problems (Table 1).

All the children in the study were assessed using an age appropriate measure of language and cognition. The cognitive tests show that the children in this sample perform below average (100) in all aspects of these tests (Table 2).

### 3.3. Relationships

**Figure 1:**
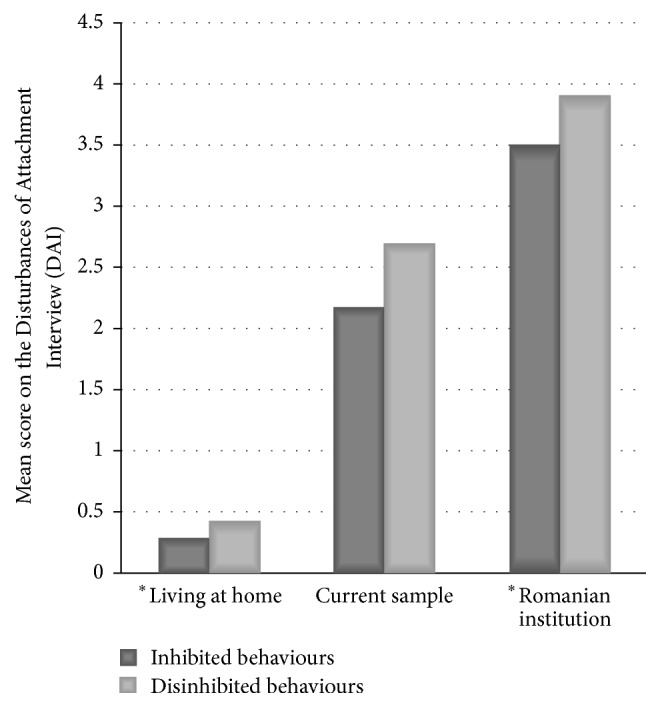
Comparisons of our sample on the Disturbances of Attachment Interview. ^*∗*^Data from [32].

Caregivers with children over the age of 12 months (*n* = 64) were also asked to complete the Disturbances of Attachment Interview (DAI). Scores are combined to identify the presence of inhibited and disinhibited behaviours (Figure 1). They can be seen compared to sample of children who had always lived at home and had never been institutionalised as well as a sample of children living in a Romanian institution.

The results of the DAI clearly show an elevated presence of inhibited and disinhibited and indiscriminate behaviours as compared to children who had never been accommodated, but not at as high level as those children living in an institution.

### 3.4. Mental Health

Because of the age range of the sample, it was necessary to use different instruments for different age groups. Mental health problems were measured using the ITSEA with foster carers of children aged 12–48 months (Table 3).

When compared to a normative sample, we see significantly higher levels of externalising behaviours in our current, in care, sample as compared to a normative sample of the same age. In addition, we see significantly less “positive” behaviour (competency and social relatedness) in the current sample compared to a normative sample.

The mental health of the children who were sampled and who were aged 2 or over was explored using the Strengths and Difficulties Questionnaire (SDQ). The results are tabulated below, firstly for the whole sample (Table 4), and then separated by gender (Table 5).

Tables 4 and 5 show that the children in our sample are showing consistently higher levels of problems than a normative sample of 3-year-old children, with significantly greater hyperactivity, peer problems, and prosocial behaviours. When separated by gender, we see a different pattern of results, with girls scoring significantly higher for emotional symptoms and hyperactivity than their female peers, while boys score significantly higher for peer problems and lower for prosocial behaviours. We see a highly significant difference, both when compared together and when separated by gender, on the level of impact which the problems these children are experiencing are having on their lives.

The Development and Wellbeing Assessment (DAWBA) was also administered in an interview with the foster carers of every child over the age of 2. Despite the DAWBA having been used in all the British nationwide surveys of child and adolescent mental health over the past decade, its use with children under the age of 5 is very much still in its infancy. It was used with 45 children in our study, of which 47% were found to have a likely diagnosis in one of the areas measured. The four most common diagnoses were Reactive Attachment Disorder/Disinhibited Social Engagement Disorder (35%), Separation Anxiety (9%), Posttraumatic Stress Disorder (7%), and Oppositional Defiant Disorder (7%).

Thirty children in our sample were scoring in the bottom 15th percentile on cognition. Their risk of showing symptoms across other domains is tabulated below (Table 6).

### 3.5. Are Foster Carers Reliable Informants?

There were weak to moderate negative correlations between the carer's level of worry and the child's language ability (rs = −0.27, *N* = 68, and *p* < 0.05); cognitive ability (rs = −0.31, *N* = 68, and *p* < 0.05), and prosocial behaviours (rs = −0.47, *N* = 38, and *p* < 0.01); that is, as the child's cognitive ability increased, the level of concern decreased. There were also significant moderate to strong positive correlations between the carer's level of worry and the child's score on dysregulation (rs = 0.51, *N* = 25, and *p* < 0.01), externalising (rs = 0.57, *N* = 25, and *p* < 0.01), conduct problems (rs = 0.34, *N* = 38, and *p* < 0.05), hyperactivity (rs = 0.40, *N* = 38, and *p* < 0.05), and peer relationship problems (rs = 0.56, *N* = 38, and *p* < 0.01); as the level of problem increased, the level of worry also increased.

Of the 30 children in the sample scoring in the bottom 15th percentile, 19 (63%) of their carers reported not being worried at all about how the child was learning to do things for himself or herself. There was no significant relationship between carer worry about those scoring above or below the 15th percentile on cognition *χ* ^2^ (2, *N* = 70) = 7.07, exact *p* = 0.13.

Although there was a positive correlation between carer worry and those children scoring above or below the 15th percentile on language (*χ* ^2^ (2, *N* = 68) = 13.06, exact *p* = 0.006), of the 23 children in the sample that scored in the bottom 15th percentile for language, 4 (17.4%) of their carers reported that they were not worried at all about how the child was making speech sounds.

The score of twelve children in the sample (aged 12–48 months) was found to be more than 3 of the ITSEA items of clinical significance. Of these, the foster carers of 2 (17%) reported not being worried at all about the child's behaviour, emotions, or relationships. There was a significant relationship between carer worry and those scoring more than 3 items of clinical significance on the ITSEA, *χ* ^2^ (1, *N* = 48) = 4.77, exact *p* < 0.05.

Carers of 45 children completed the DAWBA, and of these 45 children, 21 scored having a likely psychiatric diagnosis. Of the 21 children, carers of three (14%) reported that they were not worried at all about the child's behaviour, emotions, or relationships. There was a significant relationship between carer worry and those children identified as having a likely disorder on the DAWBA: *χ* ^2^ (9, *N* = 70) = 18.95, exact *p* = 0.041.

### 3.6. Child Engagement

We found a significant positive correlation between the child's level of engagement and their score on the cognitive measure (rs = 0.474, *N* = 56, and *p* < 0.001), with 22% of the variance in cognitive score being explained by the child's engagement in the task.

## 4. Discussion

From our results, we can see evidence in line with the previous literature showing that these children are at a much higher risk of developing cognitive and mental health problems than their peers in the general population. They are also likely to be experiencing overlapping problems across different areas of their health and development. We investigated some potential issues which arise when making these assessments and discovered that the level of worry which a carer has about a child does relate to the degree of problem the child is experiencing; however, there were still children displaying worrying symptoms which the carers are not reporting concern about, particularly when the child had a problem with cognitive functioning.

Some foster carers appeared reluctant to talk about the problems which the children were having or reported that the child had abilities that the research team did not observe whilst the child was in the clinic (these factors were observed anecdotally by the researchers and so it was too late to collect any data systematically on how were the prevalent certain behaviours). For example, carers would sometimes report that the child was saying a number of different words, yet he or she only vocalised minimally during a 3-hour assessment, despite being left alone with the carer and observed through one-way mirrors. It might be expected that foster carers would be keen for the children to remain in their care, as they have formed a relationship, and also because caring is their profession. It is therefore understandable that carers may be keen to make a good impression to those assessing the children in their care. The opposite was also observed, however, with carers sometimes reporting problems that were not witnessed by the researchers, for example, that the child “never sat still,” yet they happily sat still through a long cognitive assessment. Children may well behave differently in the clinic from how they do at home; as it is an unfamiliar environment, they are interacting with strangers and may be aware of being subject to assessment.

Most foster carers in our sample have been carers for a number of years and had had an average of 20 children in their care. We know that children in care are more likely to have problems [5] than children in the general population, and so when asked about how the children currently in their care are compared with other children, they may compare them with other foster children rather than with children from the general population. They also may normalise otherwise rare behaviours if they have cared for other children with similar difficulties. However, the same phenomenon may also help with accurate identification of certain behaviours, for example, carers with a lot of experience may be more highly attuned to hypervigilant behaviours and be better able to distinguish them from typical shyness than other adults.

Carers that are involved in the study almost never described any behaviour the child engaged in as a burden. For example, the Development and Wellbeing Assessment (DAWBA, [30]) which is used in the study involves asking carers about not only behaviours the child is doing but the impact these behaviours has on the family. Sometimes, the carers would describe extreme lengths to avoid the child getting upset, yet when asked directly about burden, they reported that this caused no problems for the family as a whole. These assessments were conducted after the child had been with the foster carer for at least 4 weeks, during which children had often shown great improvement. This may account for the lack of worry which carers report: if they have seen a child improving quickly then they will not be as worried, even if that child is still falling behind normative levels.

We expect that there may be changes to foster carers' reporting of mental health problems during the course of the randomised controlled trial (of which this study is a part) which is planned to continue until 2020. There is an extensive learning and development programme in Glasgow, run by the placement services organisation Families for Children, that starts before and after approval, continuing throughout the foster carers journey. The system around the foster carer promotes learning and development as a high priority via the Supervising Social Worker, Foster Care Reviews, and Fostering Panels.

The Scottish Government in the National Foster Care Review (2013) has also targeted foster carer training as a high priority and has recently consulted with key stakeholders, including foster carers, regarding their learning and development needs. Because of the current intense focus on infant mental health in Glasgow, we anticipate that foster carers will regard mental health of the children in their care as a key learning priority. Scottish Government is at the stage of a national implementation plan and technical standard with regard to ensuring that there is a clear training pathway, which is a mandatory requirement.

Our results found that the child's level of engagement with a task was related to how well the child performed. This may be a further example of the overlapping problems which these children are known to have; for example, those with lower cognitive scores are also perhaps more likely to have problems such as impulsivity or anxiety that interfere with their level of engagement in such tasks. For children who have suffered from early adverse experiences, being left with a stranger in a new place can increase anxiety levels, potentially more than what would be expected for a child who was not in foster care.

Many clinicians may encourage shorter assessment periods if children appear to be struggling to engage, recommending that the subsections be completed at different times for the child. This unfortunately is not always suitable for this vulnerable population: the increased levels of stress that some of these children appear to experience when being separated from their caregiver as well as the additional burden for foster carers persuade us to minimise the number of times the child has to come in for assessment.

Based on our findings, and on the literature, we would like to make some recommendations for assessing young children shortly after they enter foster care.

## 5. Lessons Learned

- Assess varying domains of a child's functioning and interpret findings as a whole.
- Identify specific issues for that child at the time, for example, problems with their mood, an understanding of which could help in the stability of the placement.
- Assessments at any time might best include consideration of the quality of current attachment relationships, thereby providing a context for understanding other assessment data.
- When a child is stressed and attachment systems are activated, reactions to unfamiliar adults and settings may be marked. When the relationship with a primary caregiver is fairly new, it may not yet provide sufficient security and comfort to help the child regulate their emotions and cope with the testing experience.
- Although the data one may obtain initially may not be representative of the child's capabilities, repeating the assessment at a later date can show which of the child's problems are decreasing as the child settles into a stable and loving home and which are more persistent and perhaps in need of more specialised intervention.
- Where possible, include data from multiple informants, using a variety of methods, and across different settings.
- Consider the environment in which the child was observed in any interpretation of findings; for example, wariness from the child in their foster placement could be indicating something different from wariness in the clinic.

## 6. Conclusions

Young children coming into foster care are at a high risk of having overlapping problems and should all be considered as potential cases with Maltreatment-Associated Psychiatric Problems (MAPP) [15].

Certain assessment issues need to be acknowledged: the children are likely to be at increased levels of stress during the assessment; the child's carer or parent may not be in the best position to give an accurate portrayal of the child's needs; while it is important to continue assessing children when they first enter care to get an accurate picture of the child's* current* needs, this may need to be repeated to establish the existence of on-going problems.

Reminding ourselves of Rowe's assertion from three decades ago: “it is the foster parents' perceptions of the seriousness of the problem that are all important” [13], it is clear that the more modern view expressed by Carter and colleagues that children should receive a multi-informant assessment is more helpful. Instead of entirely relying on the foster carers' view of the child's difficulties, we would now recommend that the child receives a holistic assessment across various domains of functioning and that that assessment may need to be repeated at a later stage, once the child has settled into placement.

We need to strive to find a way to differentiate between those children who are primarily affected by the events surrounding coming into care, for whom a nurturing placement would be likely to promote secure attachment, mental wellbeing, and movement towards rejoining a typical developmental trajectory, and those children with additional underlying problems which will require longer term support and intervention and which might adversely affect their ability to rejoin such a positive trajectory.

## Figures and Tables

**Table 1:** Scores on the Parents' Evaluation of Developmental Status (PEDS).

	No/A little	Yes
Concerns about how child talks and makes speech sounds	49 (70%)	21 (30%)
Concerns about how child understands what you say	62 (89%)	8 (11%)
Concerns about how child uses hand and fingers to do things	66 (96%)	3 (4%)
Concerns about how child uses arms and legs	63 (90%)	7 (10%)
Concerns about how child behaves	50 (71%)	20 (29%)
Concerns about how child gets along with others	59 (84%)	11 (16%)
Concerns about how child is learning to do things for him/herself	68 (97%)	2 (3%)
Concerns about how child is learning preschool skills	60 (90%)	7 (10%)

**Table 2:** Comparison of our sample to normative samples on measures of cognition and language.

		Mean scores (SD)		*t*-test
		^*∗*^Normative sample	Current sample	
*Bayley* 6–29 months (*n* = 31)	Cognitive score	103.62 (13.60)	84.5 (15.3)	*t* = 7.22, df = 1250, and **p** < 0.001
	Language score	101.92 (16.86)	86.8 (15.4)	*t* = 4.72, df = 1250, and **p** < 0.001

*WPPSI* 30–47 months (*n* = 22)	Verbal IQ	103.61 (14.32)	90.32 (14.75)	*t* = 4.19, df = 320, and **p** < 0.001
	Performance IQ	103.49 (14.94)	90.05 (15.44)	*t* = 4.06, df = 320, and **p** < 0.001
	Full scale IQ	104.19 (14.36)	88.73 (14.73)	*t* = 4.87, df = 320, and **p** < 0.001
	General Language Composite	103.59 (14.42)	91.14 (14.27)	*t* = 3.91, df = 320, and **p** < 0.001

*WPPSI* over 48 months (*n* = 16)	Verbal IQ	100.10 (13.44)	86.69 (17.16)	*t* = 3.89, df = 514, and **p** < 0.001
	Performance IQ	100.11 (14.42)	79.93 (18.86)	*t* = 5.29, df = 513, and **p** < 0.001
	Full scale IQ	99.55 (13.28)	82.27 (15.91)	*t* = 4.94, df = 513, and **p** < 0.001
	General Language Composite	100.44 (13.93)	84.42 (14.67)	*t* = 3.93, df = 510, and **p** < 0.001

^*∗*^Normative data from assessment manuals.

(i) WPPSI III administration and scoring manual, Wechsler, 2003 [37].

(ii) Bayley Scales of Infant and Toddler Developmental Third Edition, Bayley, 2006 [23].

**Table 3:** Comparisons of our sample with a normative sample on the ITSEA.

	Mean scores (SD)		*t*-test
	^*∗*^Normative sample (*n* = 1235)	Current sample (*n* = 48)	
Externalising behaviours	0.47 (0.28)	0.60 (0.43)	**t = 3.08**, **df = 1281**, and **p < 0.001**
Internalising behaviours	0.52 (0.22)	0.50 (0.31)	*t* = 0.61, df = 1281, and *p* = 0.55
Dysregulation	0.36 (0.25)	0.41 (0.29)	*t* = 1.35, df = 1281, and *p* = 0.18
Competence	1.38 (0.29)	1.14 (0.45)	**t = 5.5**, **df = 1281**, and **p < 0.001**
Maladaptive behaviours	0.11 (0.13)	0.16 (1.17)	**t = 2.58**, **df = 1281**, and **p < 0.01**
Social relatedness	1.71 (0.21)	1.53 (0.40)	**t = 5.9**, **df = 1281**, and **p < 0.001**
Atypical behaviours	0.32 (0.25)	0.32 (0.24)	*t* = 0.00, df = 1281, and *p* = 1.0

^*∗*^Data from [27].

**Table 4:** Comparing SDQ scores between our sample and a normative sample.

	Mean scores (SD)		*t*-test
	^*∗*^Normative sample (*n* = 1353)	Total current sample (*n* = 45)	
Total difficulties	9.3 (5.6)	12.13 (8.1)	**t = 3.28**, **df = 1396**, and **p < 0.005**
Emotional symptoms	1.6 (1.6)	2.07 (2.3)	*t* = 1.92, df = 1396, and *p* = 0.06
Conduct problems	2.4 (2.0)	2.38 (2.8)	*t* = 0.07, df = 1396, and *p* = 0.95
Hyperactivity	3.8 (2.5)	5.20 (3.6)	**t = 3.63**, **df = 1396**, and **p < 0.001**
Peer problems	1.6 (1.6)	2.49 (2.5)	**t = 3.59**, **df = 1396**, and **p < 0.001**
Prosocial behaviours	7.8 (1.7)	6.69 (3.4)	**t = 4.12**, **df = 1396**, and **p < 0.001**
Impact	0.3 (1.1)	1.76 (2.6)	**t = 8.19, df = 1396**, and **p < 0.001**

^*∗*^Data from http://www.sdqinfo.com/norms/UK3yearNorm.html.

**Table 5:** SDQ scores by gender compared with a normative sample.

	Mean scores (SD)				*t*-test	
	^*∗*^Normative sample		Current sample			
	Boys (*n* = 698)	Girls (*n* = 655)	Boys (*n* = 29)	Girls (*n* = 16)	Boys	Girls
Total difficulties	10	8.6	12	11.81	**p** < 0.05	**p** < 0.05
Emotional symptoms	1.6	1.6	1.69	2.75	*p* = 0.77	**p** < 0.005
Conduct problems	2.6	2.1	2.66	1.88	*p* = 0.88	*p* = 0.65
Hyperactivity	4.1	3.4	5.07	5.44	**p** < 0.05	**p** < 0.005
Peer problems	1.7	1.5	2.90	1.75	**p** < 0.001	*p* = 0.51
Prosocial behaviours	7.5	8.0	6.21	7.56	**p** < 0.001	*p* = 0.32
Impact	0.4	0.2	2.17	1.00	**p** < 0.001	**p** < 0.001

^*∗*^Data from http://www.sdqinfo.com/norms/UK3yearNorm.html.

**Table 6:** Other problems experienced by children scoring in bottom 15th percentile in cognition (*n* = 30).

	Yes	No
Bottom 15th percentile in language	**63**%	37%
DAWBA diagnosis (*n* = 20)	**55**%	45%
Scoring more than 3 items of clinical significance, ITSEA	30%	**70**%
Inhibited behaviours, DAI	**73**%	27%
Disinhibited behaviours, DAI	**76**%	24%
